# Using the MNL Model in a Mobile Device’s Indoor Positioning

**DOI:** 10.3390/biomimetics8020252

**Published:** 2023-06-13

**Authors:** Feng Xie, Ming Xie, Cheng Wang

**Affiliations:** 1 School of Information Science and Technology, Sanda University, Shanghai 201209, China; 2 School of Mechanical and Aerospace Engineering, Nanyang Technological University, Singapore 639798, Singapore

**Keywords:** mobile devices, indoor positioning, multinomial logit model, fingerprint, Wi-Fi

## Abstract

Indoor Positioning Services (IPS) allow mobile devices or bionic robots to locate themselves quickly and accurately in large commercial complexes, shopping malls, supermarkets, exhibition venues, parking garages, airports, or train hubs, and access surrounding information. Wi-Fi-based indoor positioning technology can use existing WLAN networks, and has promising prospects for broad market applications. This paper presents a method using the Multinomial Logit Model (MNL) to generate Wi-Fi signal fingerprints for positioning in real time. In an experiment, 31 locations were randomly selected and tested to validate the model, showing mobile devices could determine their locations with an accuracy of around 3 m (2.53 m median).

## 1. Introduction

Indoor Positioning Services (IPS) allow mobile devices or bionic robots to locate themselves quickly and accurately in large commercial complexes, shopping malls, supermarkets, exhibition venues, parking garages, airports, or train hubs, and know the surrounding information. In large shopping malls, museums and galleries, or hospitals equipped with Wi-Fi devices or radio-frequency identification (RFID) tags, a person with a smart phone or bionic robot will be informed of their location and given directions toward a specific place. 

Several technologies can be used for indoor positioning, including Ultra Wide Band (UWB), Wi-Fi, RFID, and Inertial Measurement Unit (IMU), etc., as reviewed by Huthaifa Obeidat et al. [1]. Each of these wireless technologies has its own advantages and limitations, and the choice of technology depends on the specific application and environmental factors. The comparison of those detection methods are shown in detailed in Table 1. Wi-Fi-based localization systems use the received signal strength indicator (RSSI) to estimate the distance between a mobile device and Wi-Fi access points. By measuring the RSSI values of signals from multiple access points, the location of the device can be estimated [2,3]. Bluetooth-based systems work similarly to Wi-Fi-based systems, where the distance between a device and multiple Bluetooth beacons is estimated by measuring the RSSI values.

It is known that at present, most major domestic shopping malls and public environments are equipped with a large number of Wi-Fi hotspots. The Wi-Fi hotspots and signal fingerprints detected by the mobile device or handheld terminal can determine the user’s location, comparable to outdoor GPS satellite positioning. 

With increasing urbanization, more large commercial buildings and working areas are being covered by Wi-Fi signals, creating opportunities for Wi-Fi-based indoor positioning services. Wi-Fi indoor positioning is a value-added service that obtains location information from end users through networks and provides corresponding location services with electronic map support.

A study by Vladimir et al. [5] systematically reviewed machine learning algorithms for indoor Wi-Fi positioning from 2019 to 2021, analyzing 64 articles. It summarized Wi-Fi positioning algorithms, finding that neural networks achieved the highest accuracy experimentally. However, many of these results were obtained in small workspaces, as Vladimir et al. mentioned.

Wi-Fi fingerprinting is a classification problem. In deep learning neural networks, optimization algorithms such as gradient descent minimize the loss function and adjust model parameters to improve classification accuracy.

In the Multinomial Logit Model (MNL), the cross-entropy loss function, also known as the negative log-likelihood, measures the difference between predicted and actual class probabilities. In neural networks, the softmax activation function typically calculates each class’s probability in the output layer, similar to the logit function in MNL. Both map input values to probability space and allow the model to predict each class’s probability.

Neural networks are complex models that learn complex data representations and capture nonlinear relationships between inputs and outputs. They are highly flexible and can achieve high accuracy in classification problems, but may require more data and computing resources to train effectively.

The MNL method may not perform as flexibly or precisely as neural networks. However, considering the trade-off between accuracy and computing resources, MNL is a small, simple, interpretable model for both binary and multi-class problems. It has a closed-form solution that can be computed efficiently and is more interpretable.

This study presented a Wi-Fi-based method for mobile device or bionic robot indoor positioning using the Multinomial Logit Model (MNL).

## 2. Background

Wi-Fi positioning technology can achieve indoor and outdoor positioning wirelessly through hotspot information. Wi-Fi-based positioning is widely used [6,7]. Currently, Wi-Fi positioning primarily uses RSS (received signal strength) through trilateration or location fingerprinting algorithms [8]. Trilateration estimates a target’s position using at least three known reference points’ distances, while location fingerprinting obtains a target’s position by comparing signal feature fingerprints required for positioning. Trilateration has lower positioning accuracy [9]. Given the same number of hotspots, location fingerprinting’s accuracy is higher than Wi-Fi trilateration, as it does not require knowing access point (AP) locations or signal channels. Currently, establishing inexpensive fingerprint maps limits location fingerprinting’s popularity and applications [10].

Location fingerprinting’s large data volume and dimensions increase the real-time online positioning computational load, affecting real-time positioning accuracy. In actual Wi-Fi indoor positioning, multipath effects, shadowing, and movement impact the RSS value each AP receives at a fixed location, reducing accuracy and increasing system complexity. First, low-resolution APs’ RSS signal variance produces noise. Second, different APs’ correlations may cause input feature redundancy. Additionally, obtaining the RSS signal’s time-varying statistics requires collecting multiple RSS samples manually at each reference point offline, limiting the large-scale deployment and practical application of real-time positioning systems.

Nicolas et al. [11] proposed Wi-Fi fingerprint indoor positioning using probability distribution comparison. Offline, Wi-Fi fingerprints containing RSSI measurements from multiple access points were collected at known building locations and stored in a radio map. Online, the current Wi-Fi fingerprint was obtained. Experiments showed a median 2.4 m error. However, high AP density and distinct Wi-Fi fingerprints at different locations may be required, with regular radio map updates.

Yang and Shao [12] proposed a Wi-Fi-based positioning technique providing meter-level accuracy using existing Wi-Fi networks without hardware changes. Transmitting and utilizing multiple Wi-Fi messages showed promising performance with low infrastructure in simulations, but potentially high computational complexity processing multiple messages. Real-world evaluations in various environments will provide insights into its effectiveness and limitations.

Hsieh et al. [13] presented Wi-Fi fingerprint-based indoor positioning using recurrent neural networks (RNN) and long short-term memory (LSTM) networks, achieving 2.5–2.7 m distance errors. RNNs and LSTMs suit modeling Wi-Fi signal changes as sensors move, but require large labeled fingerprinting datasets, hyperparameter tuning, and retraining when environments change significantly.

Deep learning methods such as RNNs and LSTMs are good at learning from sequential data such as time series. This suits modeling Wi-Fi signal changes as sensors move. However, some potential limitations are: (1) Deep learning models require large amounts of training data to perform well. Gathering and labeling fingerprinting data are expensive. (2) Hyperparameter tuning and network architecture optimization require time and expertise to achieve good results. (3) Performance may drop significantly if there are big changes to environments that models have not been trained on. Retraining or fine-tuning may be required.

The IPIN (International Conference on Indoor Positioning and Indoor Navigation) competition has aimed at evaluating real-world indoor localization systems by testing them in a realistic environment with realistic movement. In 2020, the competition included five diverse off-site tracks, each resembling real used cases and challenges for indoor positioning. The best-performing competitors obtained a third quartile of error of 1 m for the Smartphone Track and 0.5 m for the Foot-mounted IMU Track.

In 2021, the IPIN competition included three off-site tracks: Smartphone (off-site), Foot-mounted IMU (off-site), and Channel Impulse Response (off-site). The best performance for the Smartphone Track was a third quartile of error of 4.43 m; for the Foot-mounted IMU Track, it was a third quartile of error of 61.9 m, and for the Channel Impulse Response Track it was 0.0891 m, respectively (referring to the information available online at [14]).

The IPIN 2022 Indoor Localization Competition was entirely off-site and hosted six different tracks, all pedestrian-based and all indoors. The overall scores and results of IPIN 2022 Competition are shown in Table 2.

Jingxue Bi et al. [15] proposed a Wi-Fi indoor positioning algorithm based on support vector regression (SVR) optimized by particle swarm optimization (PSO), termed PSOSVRPos. SVR algorithm devoted itself to solving localization as a regression problem by building the mapping between signal features and spatial coordinates in high-dimensional space. The PSO algorithm concentrates on the global–optimal parameter estimation of the SVR model. The positioning experiment was conducted on an open dataset (1511 samples, 154 features). The PSOSVRPos algorithm could achieve positioning accuracy with a mean absolute error of 1.040 m, a root mean square error (RMSE) of 0.863 m, and errors within 1 m of 59.8%.

## 3. Model Discussion

In this paper, the Multinomial Logit Model (MNL) method used different positions’ fingerprint information points in a real-time environment as a series of optional points (universal set of alternatives). For simplicity, the most probable positioning decision points were used as a subset of this universal set. A probability-based discrete choice model is defined as a series of linear combinations based on probability and random utility:

(1) $$U_{in}=V_{in}+\varepsilon_{in}$$

The random utility function *V_in_* is composed of BSSID (Basic Service Set ID) and the level of Wi-Fi AP. $\varepsilon_{in}$ means error.

Select a series of Wi-Fi fingerprint points with the largest random utility function, and calculate their probability values,

(2) $$P_{in}=Probabilty\{V_{jn}+\varepsilon_{jn}\geq V_{in}+\varepsilon_{in},\forall i\neq j,C\subseteq S\}$$

probability function,

(3) $$P_{n}(i)=\frac{e^{V_{in}}}{\sum {}_{j\in c_{n}}e^{V_{jn}}}$$

and likelihood function:

(4) $$L*=\prod_{n=1}^{N}\prod_{i\in C_{n}}^{}P_{n}{\left(i\right)}^{y_{in}}$$

The *N* fingerprint points with the maximum probability values are used as the coordinate weights of the corresponding fingerprint points, and real-time positioning is calculated accordingly.

Wi-Fi fingerprinting appears to be a classification problem type. The multinomial logit model (MNL) and current neural networks have many similarities in classification problems.

In deep learning, neural networks model classification problems where the loss function evaluates network prediction accuracy and adjusts model parameters. In MNL, the cross-entropy loss function, also known as the negative log-likelihood, is typically used. It measures predicted and actual class probability differences, referring to the above Formulas (3) and (4). MNL assumes class probabilities are independent; cross-entropy measures the model’s probability prediction accuracy for all classes.

Unlike MNL, neural networks use various loss functions, such as mean squared error and mean absolute error. However, cross-entropy is commonly used in classification problems to measure predicted and actual probability distribution differences.

In neural networks, the softmax activation function typically calculates each output layer class’s probability, similar to the logit function in MNL. Both map input values to probability space, allowing the model to predict each class’s probability. Therefore, neural networks in deep learning and MNL have many similarities defining loss functions, especially in classification problems. The simple example below illustrates using cross-entropy loss functions in MNL and neural networks for classification.

Suppose a dataset has Wi-Fi fingerprints for different locations to classify using either MNL or a neural network. For MNL, the softmax function calculates each location class’s probability, and the cross-entropy loss function evaluates prediction accuracy. Specifically, the loss function is defined as:

(5) $$L(\theta )=-\frac{1}{n}\sum_{i=1}^{n}\sum_{j=1}^{k}y_{ij}\log p_{ij}(\theta )$$

where *θ* represents the parameters of the MNL model, *n* is the number of locations in the dataset, *k* is the number of classes (different locations), *y* is the indicator function that is 1 if the location *i* belongs to class *j* and 0 otherwise, and *P_ij_(θ)* is the predicted probability of location *i* belonging to class *j* given the parameters *θ*.

For neural networks, a similar approach uses a different loss function. The softmax function calculates each class’s probability for each image, and the cross-entropy loss function evaluates prediction accuracy. The loss function is defined as in Formula (5).

The cross-entropy loss function measures the sum of these errors over all observations. The cross-entropy loss function penalizes the model more for predictions farther from actual class probabilities. The logarithmic function amplifies the error for low-probability events, typically identifying classes correctly.

In deep learning neural networks, optimization algorithms such as gradient descent minimize the loss function and adjust model parameters to improve classification accuracy.

Overall, MNL and neural networks can classify locations similarly and use cross-entropy loss functions to evaluate prediction accuracy. Which is better depends on the specific problem.

Regarding Wi-Fi data characteristics, MNL is a simple, interpretable model for binary and multi-class problems, assuming mutually exclusive classes with an efficient closed-form solution. However, it may not be as flexible or perform as well on complex, high-dimensional datasets such as neural networks.

The MNL model is a linear model, and its coefficients directly indicate the size and direction of the influence of each feature on the output. This makes the results of logistic regression easy to understand and interpret. In contrast, many artificial intelligence algorithms are “black boxes” and their predictions are difficult to explain. Many artificial intelligence algorithms are more prone to overfitting due to their complex structure, which affects generalization ability and requires much expensive training work.

On the other hand, neural networks are complex models learning data representations and capturing nonlinear input-output relationships. Highly flexible, they suit various classification problems but may require more data and computing resources to train effectively. They can also be more difficult to interpret and diagnose, especially with more layers and neurons.

## 4. Experiments

The experiment site was located on the B1 floor of Raffles City, People’s Square, Huangpu District, Shanghai. The entire modeling and testing took two full days.

This experiment map is as follows:

To test positioning in a complex environment, a map depiction and positioning fingerprint database were established and tested on a 10,000 square meter Shanghai commercial building’s B1 floor. Adjacent to Shanghai People’s Square and directly connected to the subway hub, the densely populated B1 floor has no GPS signal coverage, but has 42 Wi-Fi APs installed.

**Figure 1:**
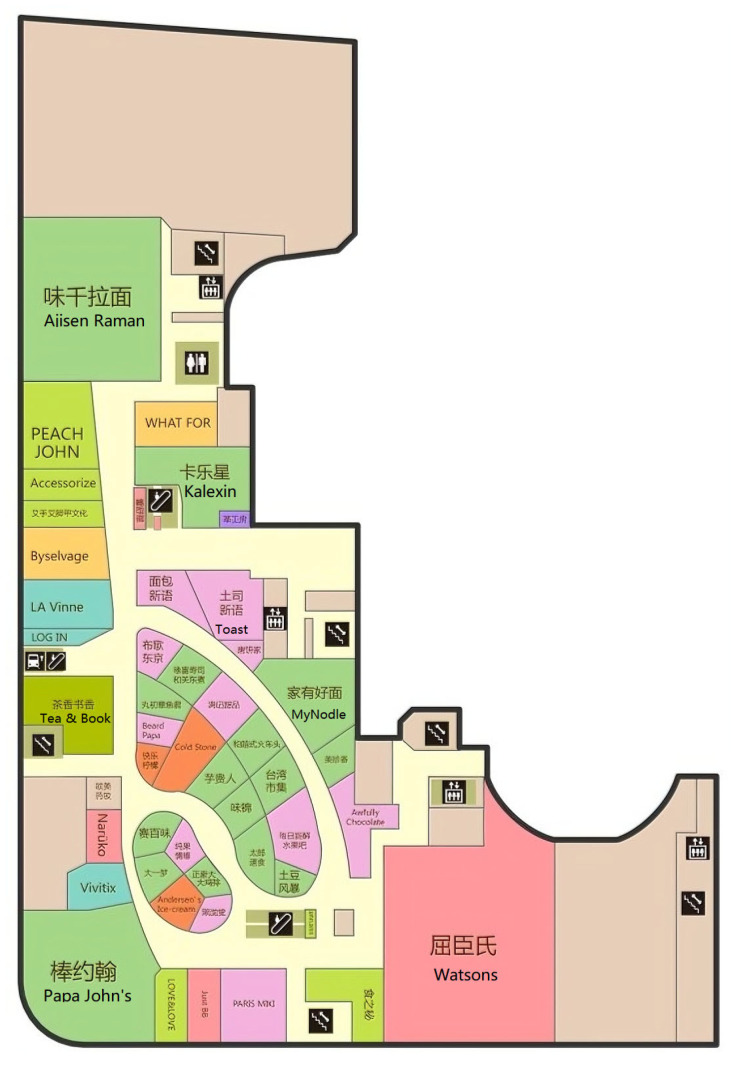
The Indoor map for experiments.

Referring to Figure 1, the background map of the experimental site used the SVG (Scalable Vector Graphics) image format. In the SVG coordinate system, the x and y axes represent 2D coordinates (the coordinate origin is in the upper left corner of the map). All geographical features, environmental locations, and coordinate information of the experimental site were pre-recorded.

The indoor positioning process based on the Wi-Fi Fingerprint method was as follows:

Step 1: We pre-surveyed and recorded the location of each position (coordinate position in SVG map) and recorded the received signal strength values of multiple Wi-Fi access points at each location.

Step 2: During the test process, the specific location and coordinate position were unknown. However, according to the actually measured Wi-Fi signal strength and compared with the information in Step 1, the classification point with the most similar signal strength pattern and its coordinates were considered as the current positioning location.

Wi-Fi hotspots provide the external conditions required for indoor positioning. Most indoor places, such as shopping malls, can use existing Wi-Fi signals. Additional hotspots may be added manually or deployed as needed where Wi-Fi signals are weak or unavailable. In some situations, indoor positioning hotspots may not connect to the Internet. Application interfaces or terminals downloaded and installed the mobile app in advance.

Several people processed the data collection over several hours. The actual positions on the digital map were recorded manually. Geographical features, such as buildings, landmarks, and infrastructure around the positioning system, may be used as reference points, mapping Wi-Fi fingerprints and their coordinates into a metric system similar to a local projected coordinate system. Comparing estimated and actual reference point locations quantifies positioning accuracy. The Maximum Likelihood algorithm calculated and displayed positions on the digital map simultaneously.

To support researching and optimizing the positioning experiment, the relevant modules were coded in Java:

- Central Management Module: basic management software (including user management, data, communication interface management).
- Central Database: using MySQL database.
- Central Communication Module: using socket communication.
- MNL Engine Module: real-time indoor positioning engine based on Maximum Likelihood probability identification.
- Central Maintenance Module: central maintenance operation, map maintenance, real-time download package, and remote/local database maintenance.

The main functions of the back-end processing center included management operations, user administration, communication interface, backup and updates, and storage of data such as maps and fingerprint information. Fingerprint information processing can be easily downloaded and used by terminals. The back-end processing center undertakes the computationally intensive and complex process of system positioning and communicates the computation results to the terminal through cloud technology so that the end user can download.

**Figure 2:**
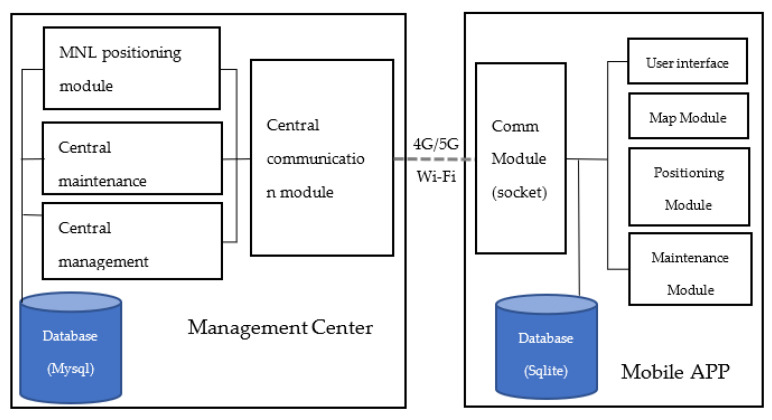
The system architecture.

The software applications installed on the mobile device were less than 50 KB, supported by 2.4 G/5.8 G wireless frequencies, across platforms and different manufacturers. The management center and mobile app (named V-IPS) are shown in Figure 2.

Another specified mobile app (named VIPS-Tester) was previously deployed for testers collecting data (including Wi-Fi raw data, and actual and calculated locations on the map of the app). The mobile app can upload the above data to the Management Center in real time.

## 5. The Dataset and Samples

The test process used a test software package to automatically collect data and stored it in the mobile device’s SQLite database according to the data format as shown in Table 3.

Real-time Wi-Fi hotspot information data were transmitted to the center in real time through the mobile phone. Transmission was guaranteed once every 5 s. The data contents included: time, Wi-Fi access point SSID, Wi-Fi access point BSSID, Wi-Fi access point LEVEL, map coordinate X, map coordinate Y, and geographic map number code.

**Figure 3:**
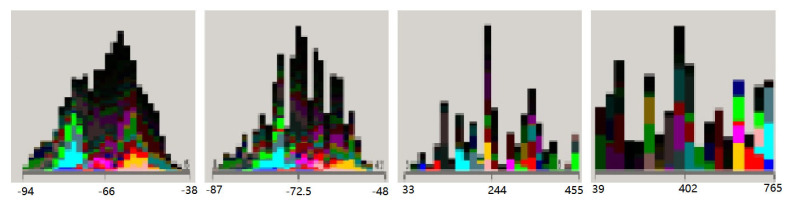
The sample distribution of datasets.

Raw fingerprint data were tidied up by calculating the maximum value (Max), minimum value (Min), average value (Mean), and standard deviation (StandDev) for each data record. It was ensured that the sample size for each fingerprint point was more than 10,000. The sample distribution of datasets was shown in Figure 3.

## 6. Model Training and Validation

The training data (with a sample size of more than 10,000) were divided into 10 parts, 9 of which were for MNL model generation and 1 for model validation and evaluation.

**Figure 4:**
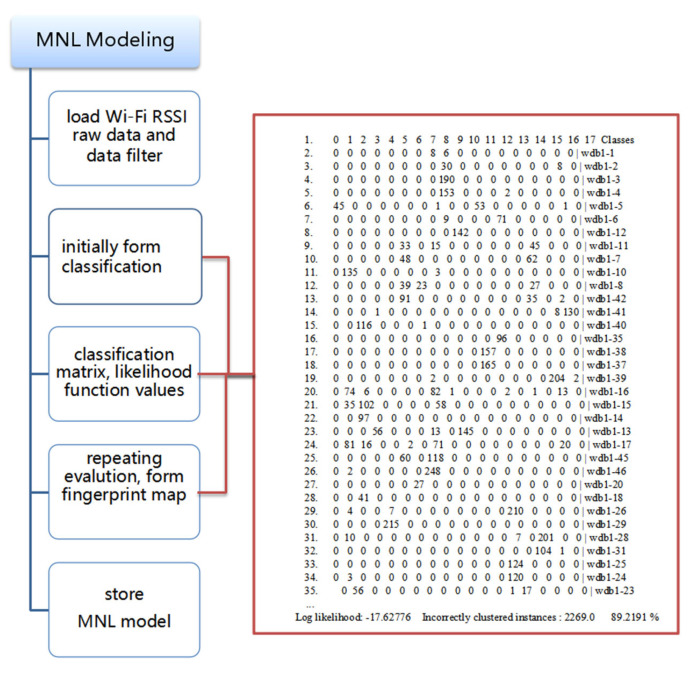
The process of the MNL modeling.

The process of the MNL modeling is shown in Figure 4.

After the first round of training was completed, the combination was shuffled, the model verification data set was changed, and the rest were calculated until 10 rounds of training were completed. The likelihood ratio test statistic, chi-squared statistic, and goodness of fit were calculated. The optimal results were stored in the central database. The collected data were processed to describe each performance according to the following:

- Accuracy: Mean error, median error, error guarantee relationship;
- Timeliness: Average positioning error within the first 15 s after arriving at a position;
- Stability: Positioning error at each position and positioning error within a certain time period (one minute) at the same position.

The error calculation was as follows:

(6) $$error=\sqrt{{\left({real}_{x}-{estimated}_{x}\right)}^{2}+{\left({real}_{y}-{estimated}_{y}\right)}^{2}}$$

**Figure 5:**
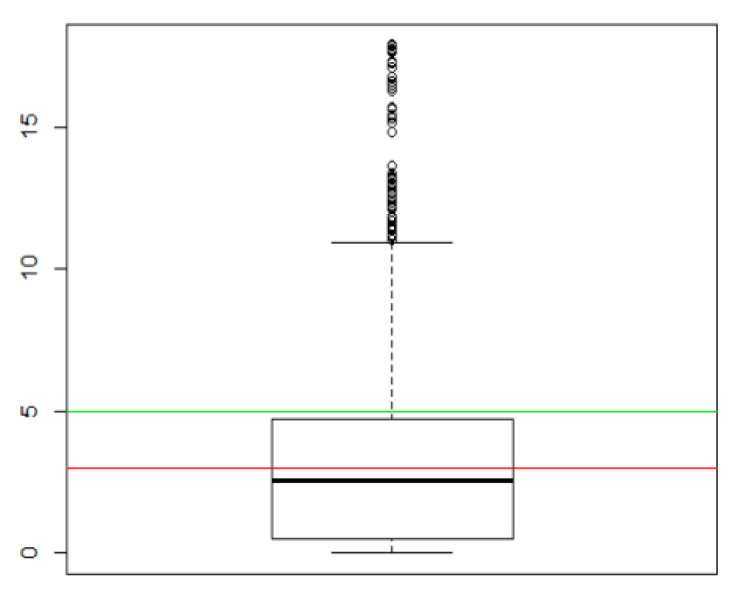
The overall accuracy of positioning.

**Figure 6:**
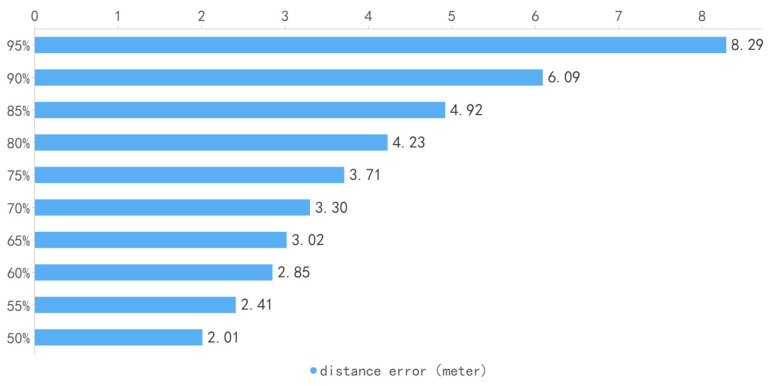
The overall distance error of positioning.

**Figure 7:**
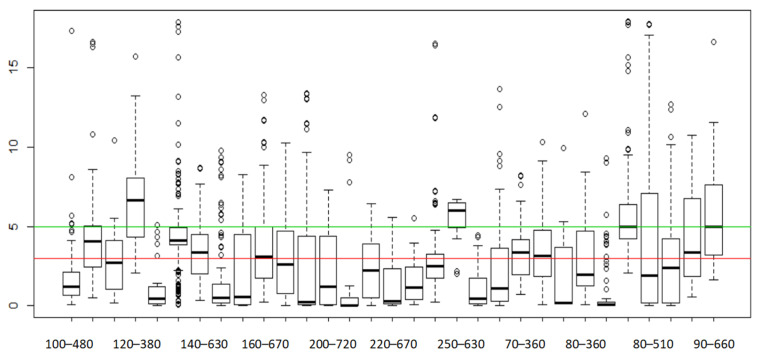
The positioning accuracy in different observed locations.

First, the difference between the actual and calculated location coordinates was calculated. Then, the error distance was calculated based on the scale of the map coordinates. In this experiment, 31 locations were randomly selected in the field, and a total of 3419 valid data points were collected. The overall performances of the tests are shown in Figure 5, Figure 6 and Figure 7, where indicate the overall accuracy of all test samples. The red line specifies the 3 m error, and the green line represents the 5 m error in Figure 5 and Figure 7.

The statistics of distance errors in the experiment are shown in Table 4. Figure 7 shows the accuracy performance of 31 locations, where the location codes (*x*-axis) represent different locations, and the *y*-axis represents the error. The red horizontal line represents the 3 m error, and the green horizontal line represents the 5 m error. The mean error (Mean) was 3.20 m, and the median error was 2.53 m (Median Value).

The studies by Hsieh et al. [13], Vladimir et al. [5], and Jingxue Bi et al. [15] were reviewed for indoor Wi-Fi positioning as in Section 2. Some results in real environments with relatively large areas were selected for comparison with the MNL model. The comparison is shown in Table 5.

Compared with the above studies, this experiment, based on the MNL model, did not achieve a very high prediction accuracy. This experiment was carried out in a complex real environment, with an experimental area of over 6500 m^2^ and 42 Wi-Fi APs installed. The software applications installed on the mobile device were less than 50 KB across platforms and different manufacturers.

Compared with some other algorithms, the MNL model was a simple linear model and could easily be used with relatively lower computational resources. Therefore, such an MNL-based experiment could be applied to a large range of areas and real scenarios. For example, in large commercial complexes or shopping malls, it could help present the passenger flow pattern and provide support for mall operations to understand hotspots and passenger behaviors. In underground parking garages, it could help vehicle owners find parking spaces quicker, or quickly find their parking vehicles.

During the experiment in this study, the size of the software application (embedding the MNL engine) installed on the Huawei Mate 10 (4 GB) mobile phone was less than 50 KB. The running memory was less than 15 MB (lower than the running memory of some general mobile input apps, such as Sogou), which was far lower than the memory standards and requirements of common mobile phones and even lower-end mobile phones on the market. The power consumption rate was about 3% when running, which was lower than the power consumption rate of WeChat (usually, the power consumption rates of messaging applications are about 5%, social applications are within 5–8%, and game and video playback applications are up to 10–15% or even higher).

Considering the cost, computational resources and user experience, 3 m accuracy may be temporarily accepted. However, in the long run, with the advancement of technology and increasing application requirements, further improvement in accuracy is the general trend, which requires continuous research and upgrading to achieve.

## 7. Conclusions and Future Work

The MNL model has been applied to indoor location positioning. As shown in the experiments, although the accuracy varied in different locations, the overall average errors were generally around 3 m (2.53 m as median values). Such accuracy would be applied to positioning for a person with a smart phone or a bionic robot’s movement in a wide indoor environment.

Some additional factors for consideration in future studies include the following:

(1) There may be bias and error in the original map and the labels of businesses and buildings on the map. It is necessary to carefully check the size, scale, and accuracy of indoor maps.
(2) If fingerprint points and sampling periods (covering different time periods) are increased, it will enrich the Wi-Fi pattern recognition database. Therefore, the positioning accuracy and stability may be improved.
(3) It is also meaningful to take into consideration the movement behaviors of people or bionic robots, and their interactive reflection with the environment in future work. It would be more conducive to assisting identification and increasing the accuracy and stability of mobile devices’ indoor positioning.
(4) MNL was adopted in this paper as it seems more interpretable. On the other hand, neural networks are more complex models that can be used to learn complex representations of the data and capture nonlinear relationships between the input features and the output classes. They are highly flexible and can be used for a wide range of classification problems, but they may require more data and computational resources to train effectively. It is meaningful to further study the trade-off between positioning accuracy and computing effectiveness. Considering the cost and user experience, with the advancement of technology and increasing application requirements, further improvement in accuracy requires continuous research and upgrading to achieve.

## Figures and Tables

**Table 1:** Positioning accuracies among different methods [4].

Type	Coverage	Accuracy	Reliability	Cost	Security	Advantage	Disadvantage
RFID	200 m	5 m	middle	low	middle	Low power consumption, wide range	Low accuracy. No communication capabilities.
Bluetooth	-	3 m	middle	high	middle	Small device size	Expensive, poor stability and easily affected by noise.
Wi-Fi	35 m	2–10 m	middle	low	middle	Wide applications; simple and cheap hardware	The workload of collecting data is large, and the location setting of the AP is cumbersome.
UWB	20 m	0.10 m	middle	middle	high	Undisturbed, low energy consumption	Short range, high hardware cost
Infrared		5–10 m	low	high	high	Suitable for use in open rooms	Easily blocked by objects or walls, short transmission distance and cost is high.

**Table 2:** Overall scores and results of IPIN 2022 competition.

Track/Team	Members	3rd Quartile
Track 2: Camera		
1: CamLoc	Institute of Computing Technology, Chinese Academy of Sciences; Beijing University of Posts and Telecommunications	2.1 m
2: SZUSCRI	Shenzhen University Smart City Research Institute	3.2 m
Track 3: Smartphone		
X-lab	Nanjing University of Aeronautics and Astronautics, College of Automation	30.1 m
Leviathan	School of Electronic and Information Engineering, Beihang University	39.8 m
Track 4: Foot-mounted IMU		
SmartFoot	Beihang University	>3 × 15 m
X-lab	Nanjing University of Aeronautics and Astronautics, College of Automation	>3 × 15 m
Track 6: Smartphone on vehicle		
1: WHU-GD	GNSS Center, Wuhan University, China; Gaode Map Company, China	14.7 m
2: team708	School of Software Engineering, Beijing Jiaotong University	22.3 m
Track 7: CIR in warehouse		
1: ISCAS	Institute of Software, Chinese Academy of Sciences; Tencent Inc.	0.2 m
1: imec-WAVES	imec-WAVES	0.2 m
Track 8: 5G in open-plan office		
1: Mobile	Institute of Software, Chinese Academy of Sciences	0.8 m
2: DYS-BUPT	Beijing University of Post and Telecommunication, China	22.3

**Table 3:** An example of the raw test data format (unit: meter).

Time (hour:min:sec)	real_x	real_y	esti_x	esti_y	Distance Error
16:27:35	78.75	493.84	42.62	516.68	5.09
16:27:40	78.75	493.84	43.92	516.72	4.96
16:27:46	78.75	493.84	43.92	516.72	4.96
16:27:58	81.58	509.41	44.04	516.82	4.56

Where: time: time of data collection HH:MM:SS; real_x: x coordinate of the actual location; real_y: y coordinate of the actual location; esti_x: the x coordinate of the location calculated by MNL; esti_y: the y coordinate of the location calculated by MNL; distance errors: the distance difference between the actual location and one calculated by MNL.

**Table 4:** The statistics of distance errors.

Distance Errors	Unit: Meter
Median	2.53
Mean	3.20
RMSE (root-mean-square error)	1.73
90th percentile	8.29
75th percentile	3.71

**Table 5:** The comparison of results.

Year	APs Used	Reference Points Used in Offline Phase	Size of Experimental Area (m²)	Algorithms	Mean Distance Error (m)	Other Metrics Reported in the Study	References
	42	85	6500	MNL	3.20	RMSE = 1.73 m	This Paper
2023	-	-	-	PSOSVRPos	1.040	RMSE = 0.863 m errors within 1 m of 59.8%	Jingxue Bi et al. [15]
2020	4	264	112	RF	1.68		Vladimir et al. [5]
2020	4	10	169	CNN	0.98		Vladimir et al. [5]
2020	6	112	60	capsnet	0.68		Vladimir et al. [5]
2020	8	133	512	Deep Fuzzy Forest	1.36	RMSE = 1.79 m	Vladimir et al. [5]
2020	6	50	60	RF Bernoulli distribution		RMSE = 2.50 m	Vladimir et al. [5]
2020	25	240	315	RF Co-forest	2.44		Vladimir et al. [5]
2020	195	300	800	DNN HMM	1.22	RMSE = 1.43 m	Vladimir et al. [5]
2020	4	236	1148	BPNN GA-PSO	0.215		Vladimir et al. [5]
2020	10	102	568.4	LSTM LF-D	1.48		Vladimir et al. [5]
2020	30	353	2750	LSTM LF-D	1.75		Vladimir et al. [5]
2020	520	993	293	CNN,LSTM	3.65		Vladimir et al. [5]
2020	5	22		DNN			Vladimir et al. [5]
2020	N/A	157	5500	RNN DL	3.05	std = 2.818	Vladimir et al. [5]
2020	N/A	40	UJI Library	CNN			Vladimir et al. [5]
2018	N/A	N/A	N/A	RNN	2.5–2.7		Hsieh et al. [13]

## Data Availability

Not applicable.
